# Photomodulation alleviates cellular senescence of aging adipose-derived stem cells

**DOI:** 10.1186/s12964-023-01152-x

**Published:** 2023-06-19

**Authors:** Tao Zhang, Yuqian He, Xin Shu, Xiaoyu Ma, Jiaqi Wu, Zuoqin Du, Jin Xu, Ni Chen, Jingcan You, Yaofang Liu, Tian Li, Jianbo Wu

**Affiliations:** 1 Department of Pharmacology, School of Pharmacy Drug Discovery Research Center of Southwest Medical University, and Laboratory for Cardiovascular Pharmacology, Southwest Medical University, Luzhou, Sichuan China; 2 Key Laboratory of Medical Electrophysiology of Ministry of Education, Collaborative Innovation Center for Prevention and Treatment of Cardiovascular Disease of Sichuan Province, Southwest Medical University, Luzhou, China; 3 Metabolic Vascular Disease Key Laboratory of Sichuan Province, Luzhou Municipal Key Laboratory of Thrombosis and Vascular Biology, Luzhou, 646000 Sichuan China; 4 Department of Reproductive Technology, The Affiliated Hospital of Southwest Medical University, Luzhou, 646000 Sichuan China; 5 Drug Discovery Research Center, Southwest Medical University, Luzhou, 646000 Sichuan People’s Republic of China

**Keywords:** Photomodulation, Adipose tissue derived stem cells, Senescence, In vitro-passage, Opsin3

## Abstract

**Background:**

Mesenchymal stem cells (MSCs) therapies are emerging as a promising approach to therapeutic regeneration. Therapeutic persistence and reduced functional stem cells following cell delivery remain critical hurdles for clinical investigation due to the senescence of freshly isolated cells and extensive in-vitro passage.

**Methods:**

Cultured adipose-derived stem cells (ASCs) were derived from subcutaneous white adipose tissue isolated from mice fed a normal diet. We performed senescence-associated-β-galactosidase (SA-β-gal) staining, real-time PCR, and Westernblot to evaluate the levels related to cellular senescence markers.

**Results:**

The mRNA expression levels of senescence markers were significantly increased in the later passage of ASCs. We show that light activation reduced the expression of senescent genes, and SA-β-Gal in all cells at passages. Moreover, the light-activated ASCs-derived exosomes decrease the expression of senescence, and SA-β-Gal in the later passage cells. We further investigated the photoreceptive effect of Opsin3 (Opn3) in light-activated ASCs. Deletion of Opn3 abolished the differences of light activation in reduced expression of senescent genes, increased Ca ^2+^ influx, and cAMP levels.

**Conclusions:**

ASCs can undergo cellular senescence in-vitro passage. Photomodulation might be better preserved over senescence and Opn3-dependent activation in aged ASCs. Light-activated ASCs-derived exosomes could be served as e a new protective paradigm for cellular senescence in-vitro passage.

Video Abstract

**Supplementary Information:**

The online version contains supplementary material available at 10.1186/s12964-023-01152-x.

## Introduction

Accumulating evidence has indicated the therapeutic value of mesenchymal stem cells (MSCs) in regenerative medicine. For in-vitro culture, MSCs must be expanded over replicative, multiple population doublings to obtain an available and sufficient number of cells for administration [1, 2]. Previous studies have primarily shown the different conditions of population doublings from the initial MSCs passage to senescence, which is linked to loss of the replication and differentiation capability [3–7].

Cellular senescence occurs due to multiple factors such as in vitro cell aging, telomere attrition, irradiation, oncogene activation, oxidative damage, and mitochondrial dysfunction. Several aging-related experimental therapeutic approaches, such as senolytics, antioxidants, and exercise, have delayed the onset of cellular senescence [8, 9].

Understanding the molecular mechanism involved in the aging-associated deterioration of stem cell function is crucial in developing effective new therapeutic methods. Photomodulation involves sending specific light packets that can be absorbed by photoreceptors or molecules associated with the mitochondria of cells. Numerous studies have demonstrated the advantages of photomodulation as a promising physical approach in various pathologies, particularly for cell and tissue regeneration. In our recent in vitro ASCs culture study, light treatment was found to significantly increased cell proliferation compared to non-light treatment [10]. In this study, we tested the hypothesis that photomodulation could alleviate cellular senescence in ASCs in vitro. The results suggest that photomodulation may serve as a preventive measure against cellular senescence during in-vitro passage of ASCs.

## Materials and methods

### Animals

6–8 weeks old C57BL/6 J mice were obtained from the Chongqing Medical University, Chongqing, China. All protocols for animal use were reviewed and approved by the Animal Care Committee of Southwest Medical University in accordance with Institutional Animal Care and Use Committee guidelines.

Mice with floxed Opn3 allele and mice expressing Cre recombinase driven by *adiponectin* (adipoq-Cre) were obtained from Cyagen Biosciences (Suzhou, China). Opn3 ^fl/fl^ was mated with adipoq-Cre after being backcrossed for 10 generations into the C57BL/6 J background. Opn3^fl/fl^ mice were mated with Opn3 ^fl/fl^ adipoq-Cre( ±) to generate Adioopn3 -/- mice. Cre-negative littermates were used as WT controls.

### Culture of ASCs

Mouse adipose-derived stem cells (ASCs) were isolated from inguinal subcutaneous fat of C57BL/6 mice that were fed normal chow (NFD). The cells were cultured as described previously [10]. Briefly, subcutaneous adipose tissues were digested with collagenase type 1 (Sigma-Aldrich, St. Louis, MO, USA) in PBS (Phosphate-buffered saline) by incubation in a shaker at 37 °C for 30 min. Cells were suspended in complete medium made of DMEM/F12 (Dulbecco modified Eagle medium) medium supplemented with 10% fetal bovine serum (FBS) and 1% penicillin and streptomycin. Cells were cultured in 37 °C at 5% CO2 and 20% O_2_ incubator. Flow cytometry is used to identify the ASCs with surface markers as described previously by us [10, 11]. Briefly, ASCs are positive CD29 (BD Pharmingen), CD90 (BD Pharmingen), and CD105 (Biolegend) in a normal conditional and light treatment. Additionally, ASC are negative for CD31 (Biolegend) and CD45.( Biolegend). ASCs at passage 3–10 were used in all the experiments.

### Photomodulation

Cultured ASCs were transferred into a sterile syringe and subjected to light treatment for 30 min using a CellRegena Device (HarmonyRegena CO., Ltd, China) [10, 11]. The syringe was then placed into the device and rotationally activated by light-emitting diode (LED) light. This device integrates monochromatic lights of three different wavelengths, including 575–595 nm (5–20 mW), 630–635 nm or 660–670 nm (10–100 mW) and/or 510–540 nm (10–60 mW) of monochromatic light.

After light-treated for 30 min, ASCs were prepared for subculture every 10 days up to passage 9 and culture media were completely renewed every 3 days. A second set of cells was subjected to non-light treatment for 30 min as control group, which cultured ASCs were transferred into a sterile syringe without light treatment.

### Exosome purification and characterization

Exosomes were isolated from cultured ASCs using an PS affinity MagCaptureTM Exosome Isolation Kit (Wako Life Sciences; Richmond, VA, USA). The cultured medium was collected and performed a preliminary centrifugation 300 g for 10 min. The supernatant. was then subjected to centrifugation at 3000 g for 30 min, and then passed through a 0.22 μm filter, and transferred to an ultrafiltration concentration tube Vivaspi Turbo 15 for concentration. The concentration of exosomes were evaluated by Nano Sight NS3000.

### Quantitative real-time PCR

ASCs were collected and total RNA was extracted using TRIzol reagent (Invitrogen, Carlsbad, CA, USA). RNA samples were pre-treated with deoxyribonuclease I (Invitrogen Life Technologies, Carlsbad, CA, USA), and a SuperScript kit (Invitrogen Life Technologies, Carlsbad, CA, USA) was used to synthesize cDNA according to the manufacturer’s recommendations. qRT-PCR was analyzed using miScript SYBR Green PCR Kits (Qiagen). Levels of cell cycle markers (p16, p21, and p53) mRNAs were determined by ABI PRISM 7700 cycler (Applied Biosystems, Foster City, CA). Each sample was analysed in duplicate with ribosomal 18S RNA as an internal control. Using telomeric primers, primers for the reference control gene (mouse 36B4 single copy gene), qPCR settings were performed as previously described [12]. The relative telomere length was shown as a T/S ratio indicative from the single copy gene. All fold changes in gene expression were determined using the 2 − ΔΔCT method. The values are presented as the mean ± SEM. All primers are listed in Supplemental Table 1.

### Immunoblotting

ASCs lysates were prepared, and equal amounts of protein were subjected to SDS-PAGE and transferred to polyvinylidene difluoride membranes by electroblotting. After blocking, the membranes were incubated with antibodies directed against Opn3 (Cell signaling), p16 (Abcam), p21(Servicebio), and p53 (Abcam),. Secondary antibody was horseradish-peroxidase (HRP)-conjugated goat IgG raised against IgG (Santa Cruz Biotechnology). Blots were developed with ECL substrate (Pierce).

### Senescence-Associated-β-Galactosidase (SA-β-gal) Assay

The SA-*β*-gal activity was measured using Senescence Assay Kit (ST429, Beyotime) based on the manufacturer’s instructions. Briefly, cells were incubated in ONPG at room temperature for 12 h then stained with the Staining Mixture at 37 °C without CO_2_ overnight. Subsequently, cells were observed and visualized under a light microscope (Zeiss HAL 100). The values were normalized to total protein of cell lysates assessed with a bicinchoninic acid (BCA) protein assay (Pierce).

### Intracellular Ca^2+^ concentration

To evaluate the changes in intracellular calcium concentrations, a calcium-sensitive fluorescence indicator, Fluo-8, AM (AAT Bioquest, Sunnyvale, CA, USA), was used to stain the cells according to the manufacturer’s instructions. Briefly, Cells were washed in serum- and phenol red-free DMEM containing 4 μM Fluo-8 AM plus 0.08% Pluronic F127 (AAT Bioquest, Sunnyvale, CA, USA) for 20 min at 37 °C, 5% CO_2_ to load the dye into the cells. 25 mM Probenecid (AAT Bioquest, Sunnyvale, CA, USA) solution was used to reduce leakage of intracellular dye. The fluorescence of Fluo-8 AM was excited at the wavelength of 490 nm and measured using a fluorescence microplate reader (Cytation 5, USA). Three independent experiments were averaged.

### cAMP level

Under the condition of non-light treatment or light treatment for 30 min, wild-type and Adipopn3-deficient ASCs were seeded into six-well platelets 3 × 10^5^ cells per well, grown for 24 h, then serum-starved overnight in DMEM containing 0.1% fatty acid-free bovine serum albumin, and stimulated with 5 nM S1P and/or 1 µM forskolin (Bio-Techne, Abingdon, UK, 1099/10) or were mock-stimulated, for 10 min at 37 °C. The medium was aspirated, and supernatant was collected by centrifugation at 700 × g for 5 min. cAMP levels were measured using the general cAMP ELISA kit (Immuno clone Biosciences co., USA) according to the manufacturer’s instructions.

### Statistical analysis

Data are presented as the mean ± SEM of triplicate experiments. Experimental groups were compared by the two-tailed Student’s t-test or.one-way analysis of variance (ANOVA). All analyses were performed with SPSS software (version 24.0 for Windows; Armonk, NY, USA), and a level of *P* < 0.05 was defined as indicative of statistical significance.

## Results and discussion

### Light activation prevents the onset of senescence in ASCs

**Fig. 1:**
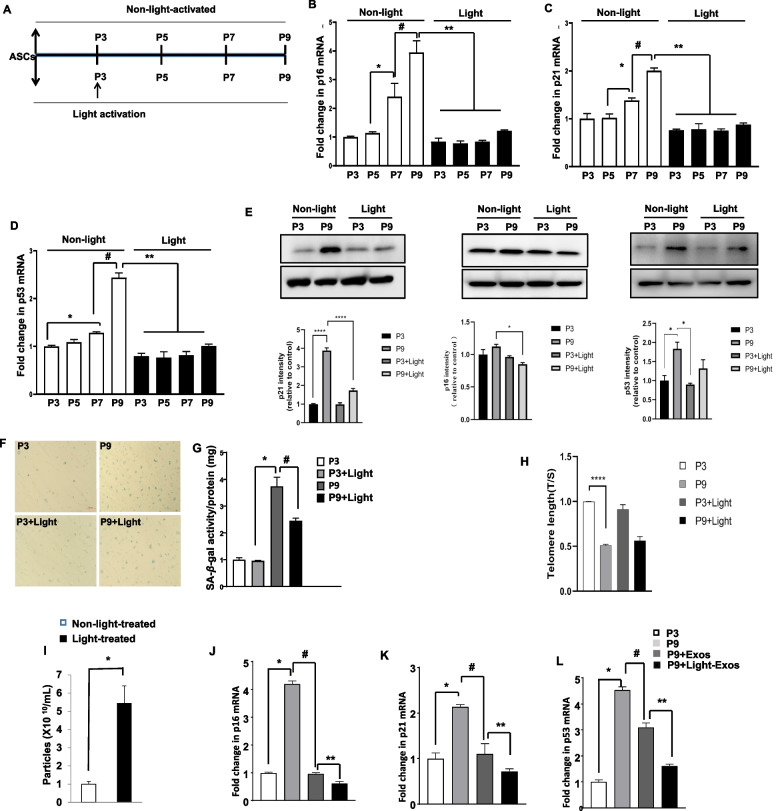
Light activation prevents the onset of senescence in ASCs. **A** Schematic timeline of ASCs transplantation model. Light treatment was performed at passage 3 (P3). After light activation for 30 min, WT-ASCs were prepared for subculture every 10 days up to passage 9 and culture media were completely renewed every 3 days. A second set of cells was subjected to non-light activation for 30 min as control group. The expression of p16 (**B**), p21 (**C**), and p53 (**D**) was evaluated by qPCR from P3-P9 in ASCs from normal diet fed C57BL/6 J mice. Significance was calculated using an ordinary one-way ANOVA. Data shown as mean ± SEM for three independent experiments. **E** p16, p21, and p53 protein levels measured by Western blotting at P3 and P9 from non-light and light-treated ASCs. The graph corresponds to the adjacent blots and represents densitometric analyses of 3 individual samples. *P < 0.05, ^****^P < 0.001. Significance was analyzed using an ordinary one-way ANOVA. Data shown as mean ± SEM. **F** Representative images of SA-β-galactosidase-positive cells. **G** Senescence was evaluated in terms of SA-β-galactosidase activity and expressed as the ratio of cells protein (mg). Scale bar: 50 μm. **H **Telomere length shown as T/S ratio evaluated by qPCR. ^****^p < 0.001, compared to P3. **I** The relative concentration of exosomes from from ASCs with indicated treatments. **J-L** Light-activated-ASCs-Exos presented a marked efficacy in the prevention of cellular senescence**.** 1 × 10^8^/mL of exosomes were added in the cultured ASCs for 24 h as indicated treatment. The expression of p16 (**J**), p21 (**K)**, and p53 (**L**) was assessed by qPCR. *n* = 3–5 per group. Data shown as mean ± SEM. *P < 0.05; **P < 0.01; ^#^*P* < 0.05; ^##^*P* < 0.01

We evaluate the cellular senescence in-vitro passage of cultured ASCs derived from the normal-fat diet C57BL/6 J mice. Following the schematic timeline in the experimental model (Fig. 1A), we observed that the in vitro increased passage number of ASCs led to significantly greater expression in the cell cycle gene p16 and p21 as well as p53 at passages 5, 7, and 9 (P5, P7, and P9) (Fig. 1B-D). Conversely, the levels of senescence genes were the same regardless of P3-P9 when ASCs were activated with light at P3. The levels of p16, p21, and p53 expression were further evaluated by Westernblotting. As shown in Fig. 1E, the levels of p16, p21, and p53 were markedly increased in ASCs at P9 compared with that at P3. In contrast, light-treated caused a significant decrease at the levels of p16, p21, and p53 in ASCs at P9 compared with that non-light-treated P9.

Light activation also decrease in SA-β-galactosidase activity at P9 of ASCs (Fig. 1F, G). These findings demonstrated that photomodulation effectively reduced the expression of the critical cellular senescence markers and SA–β-gal for the cellular senescence in-vitro passage. To investigate the cellular senescence associated with short telomeres, we evaluated the telomere lengths in vitro passage culture (Fig. 1H). Telomeres were significantly shorter at P9 than P3 in non-light-treated group. However, we observed the light treatment exhibited a slight change in the telomere length. Future studies are necessary to define the relationship more precisely between cellular senescence and telomeres.

We explore the effects of photomodulation on exosome secretion. ASCs at P3 were treated with light for 30 min. The concentration of light-treated ASCs-Exos was approximately a fivefold increase in the number of exosomes secretion compared with non-light-treated ASCs-Exos (Fig. 1I). Aging ASCs were treated with ASCs-derived exosomes for 24 h. P9 treated with ASCs-Exos showed a significant downregulation in the mRNA levels of p16, p21, and p53. Notably, the light-activated ASCs-Exos group presented marked lower levels than the non-light-activated ASCs-Exos group (Fig. 1J-L), indicating that light-activated-ASCs-Exos exhibited significant efficacy in the prevention of cellular senescence.

### Opn3 Is required for light activation-dependent regulation of cellular senescence

**Fig. 2:**
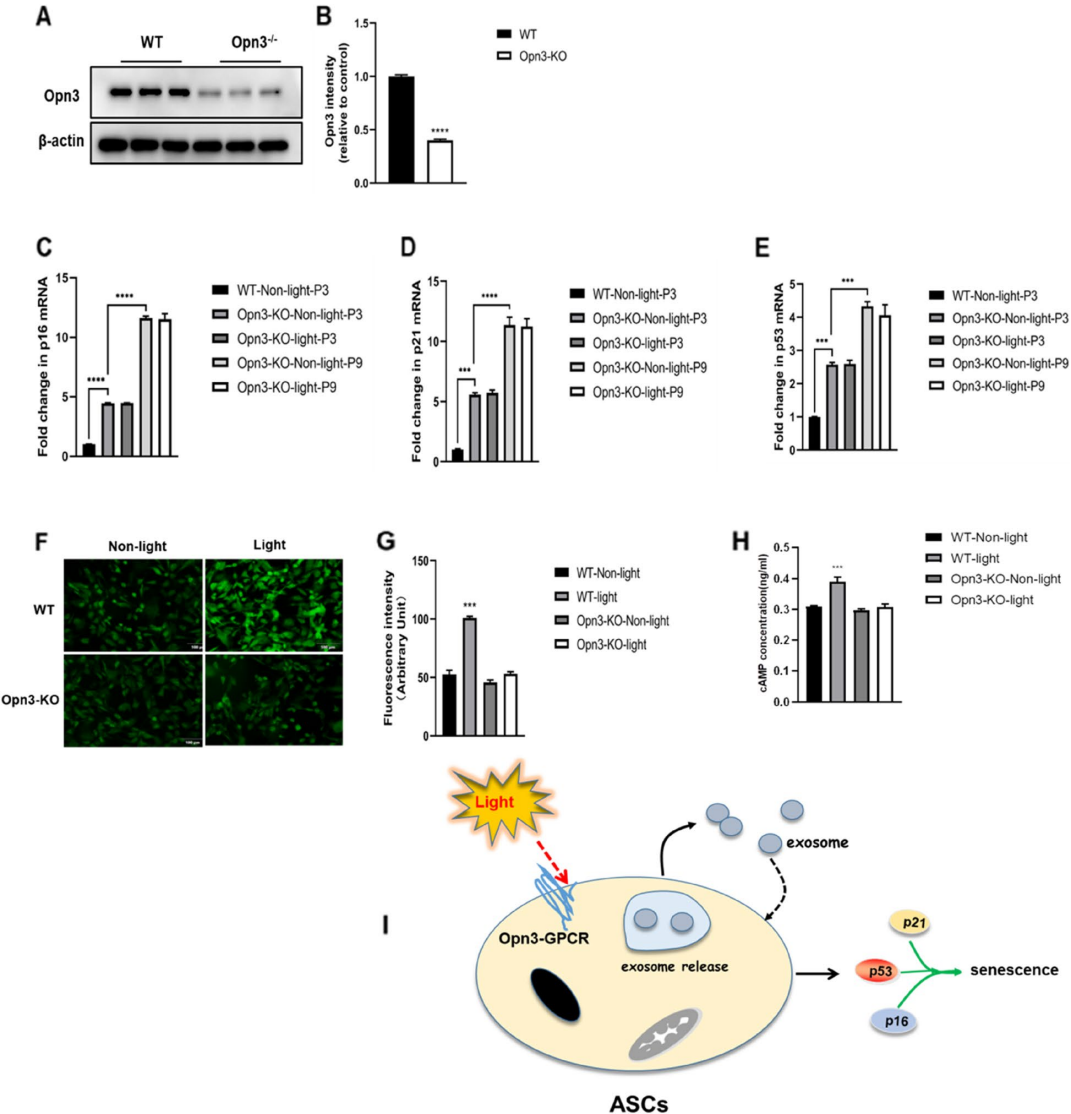
Opn3 Is required for light activation-dependent regulation of cellular senescence. **A** Westernblotting analysis confirmed the Opn3 expression in cultured WT- and Opn3^−/−^-ASCs. **B** Densitometric analysis of Opn3 band. **C-E** After light activation for 30 min, ASCs were prepared for subculture every 10 days up to passage 9 and culture media were completely renewed every 3 days. A second set of cells was subjected to non-light activation for 30 min as control group. The expression of p16 (**C**), p21 (**D**), and p53 **(E**) was evaluated by qPCR at P3 and P9 in either WT-ASCs or Opn3^−/−^-ASCs. Significance was analyzed using an ordinary one-way ANOVA. Data shown as mean ± SEM for three independent experiments. **F** Intracellular calcium concentration was measured by using Fura fluorescence in ASCs as indicated treatment. Light activation was performed at passage 3 (P3). **G** Quantification of relative fluorescence intensity is shown. **H** Light activation show Opn3-dependent elevation of cAMP in ASCs ass indicated. Data shown as mean ± SEM for three independent experiments. *P < 0.05; ***P < 0.01; ****P < 0.01. **I** Schematic illustration of showing that light activation alleviates the cellular senescence in-vitro passage by involving increased Ca ^2+^ influx and cAMP levels in cultured ASCs

It is important to note that photomodulation exhibits a dominant efficacy in preventing cellular senescence. Opsins (Opn3 or encephalopsin) are a family of light-activated, retinal-dependent, G protein-coupled receptors (GPCRs) that are identified in adipose tissues [13]. To study the functions of Opn3 expressed by ASCs during in-vitro passage of cultured ASCs, we compared the senescent gene expression of ASCs isolated from WT and Opn3^−/−^ mice. Westernblotting analysis confirmed that Opn3 expression was markedly decreased in Opn3^−/−^-ASCs as compared with WT-ASCs (Fig. 2A-B). At the P3, compared to WT-ASCs, Opn3-deficient ASCs showed significantly increased mRNA levels of p16, p21, and p53 (Fig. 2C-E), suggesting that under physiological conditions, the dominant effect of basal Opn3 expression by ASCs is anti-senescence. Moreover, we did not observe the inhibitory effects of light treatment at passage 3 in Opn3^−/−^-ASCs. Similarly, the increased levels of mRNA p16, p21, and p53) were much greater at P9 than at P3, and light treatment had no effects on the expression in Opn3^−/−^-ASCs. We showed that Opn3 is required for light-dependent regulation of cellular senescence biomarkers, suggesting that light activation likely modifies ASCs-derived exosomes and might affect cellular senescence in an autocrine/paracrine manner.

Previous studies have shown that exosome release is regulated by a calcium-dependent mechanism [14, 15]. We used Fura fluorescence to measure cytoplasmic calcium concentration alteration in the cultured ASCs. Our data showed that light activation significantly increased the Ca^2+^ concentration in WT-ASCs compared with the non-light-activated ASCs group. However, we did not find any changes in Ca^2+^ concentration in Opn3^−/−^-ASCs (Fig. 2F, G). These data indicated that photomodulation stimulates exosomes release of ASCs is associated with increased intracellular calcium concentration and Opn3-dependent manner. Future studies need to be conducted to look into the effect of photomodulation on ASCs-Exos and miRNA signaling and other proposed mechanisms.

Opsins are coupled with G proteins signal via a pathway involving intracellular second messenger, adenosine 3′, 5′-cyclic monophosphate (cAMP) [16]. Assessment of cAMP from ASCs lysates revealed that light activation showed significantly elevated cAMP in WT-ASCs compared with the non-light-activated ASCs group. However, we did not find any changes in cAMP level from Opn3^−/−^-ASCs in the absence or presence of light activation (Fig. 2H), supporting the hypothesis that Opn3 is required for a light-dependent regulation of cellular signaling and senescence.

## Supplementary Information

**Additional file 1:** **Supplementary Fig. 1.** Light treatment prevents the onset of senescence in ASCs A-C. The expression of p16, p21, and p53was assessed by qPCR at P3 and P5 AQin ASCs from high fat dietfed C57BL/6 J mice. NFD-P3 as compared a control. E Representative images of SA-β-galactosidase-positive cells. F Senescence was evaluated in terms of SA-β-galactosidase activity and expressed as the ratio of cells protein. Scale bar: 50 μm.

**Additional file 2: Supplemental Table 1.** Primers used in qPCR.

## Data Availability

The data generated or analyzed during this study are included in this article, or if absent are available from the corresponding author upon reasonable request.

## Abbreviations

ASCs: Adipose-derived stem cells
cAMP: Cyclic adenosine monophosphate
GPCRs: G protein-coupled receptors
MSCs: Mesenchymal stem cells
ND: normal diet
WT: Wild type
