# Supplementary material for: Photomodulation alleviates cellular senescence of aging adipose-derived stem cells
Source: Cell Commun Signal. 2023 Jun 19;21:146. doi: 10.1186/s12964-023-01152-x (PMC10278352; doi:10.1186/s12964-023-01152-x)
Supplement: Supplementary file 2 — Additional file 1: Supplementary Fig. 1. Light treatment prevents the onset of senescence in ASCs A-C. The expression of p16, p21, and p53was assessed by qPCR at P3 and P5 AQin ASCs from high fat dietfed C57BL/6 J mice. NFD-P3 as compared a control. E Representative images of SA-β-galactosidase-positive cells. F Senescence was evaluated in terms of SA-β-galactosidase activity and expressed as the ratio of cells protein. Scale bar: 50 μm. [file 12964_2023_1152_MOESM1_ESM.pptx]

## Slide 1
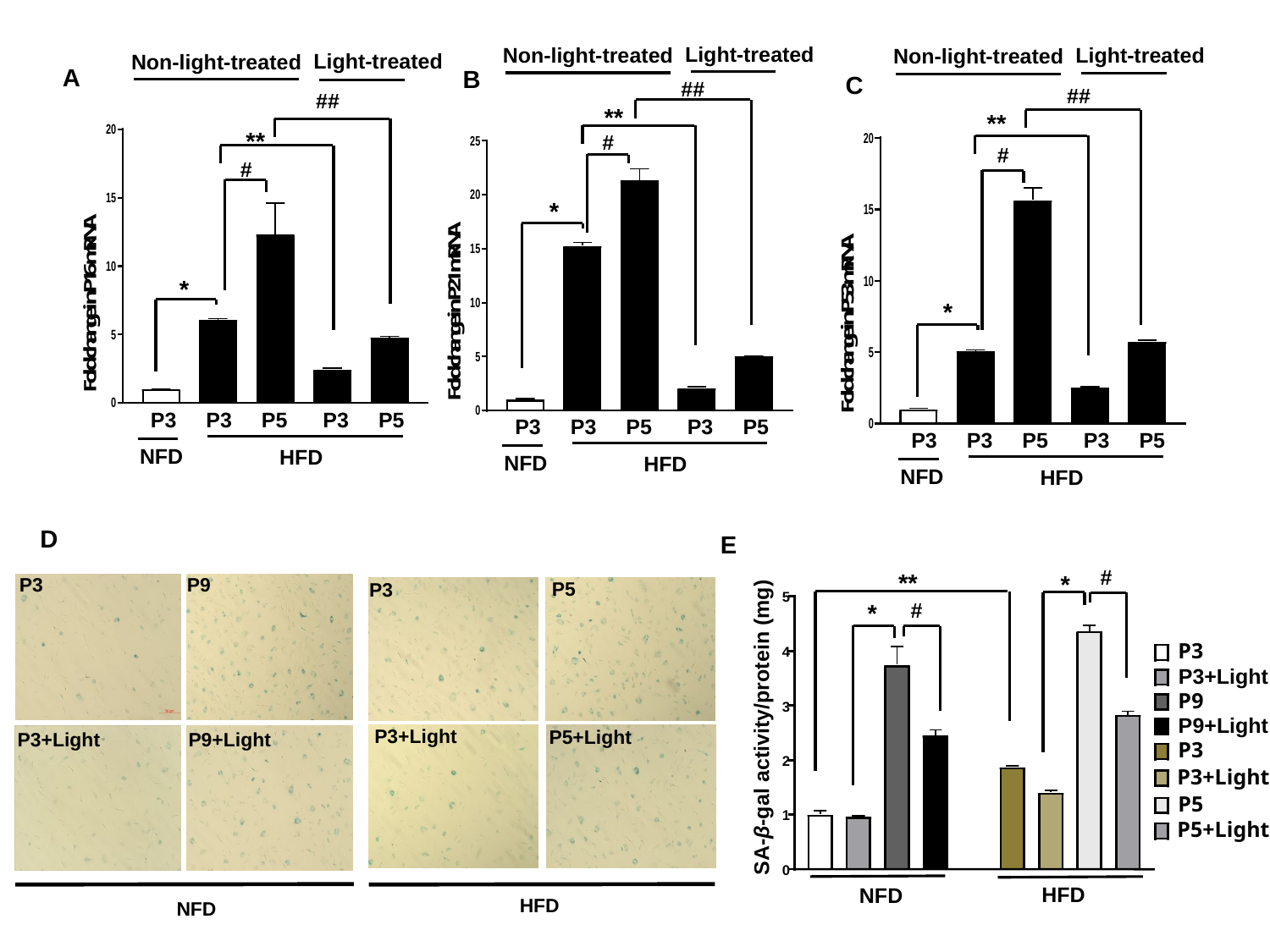

Light-treated
Non-light-treated
Light-treated
Non-light-treated
Light-treated
Non-light-treated
A
B
C
##
##
##
**
**
**
#
#
#
*
*
*
P3 P3 P5 P3 P5
P3 P3 P5 P3 P5
P3 P3 P5 P3 P5
NFD
HFD
NFD
HFD
NFD
HFD
D
E
#
**
*
P3
P9
P3+Light
P9+Light
NFD
P5
P3
P3+Light
P5+Light
HFD
5
#
*
P3
4
P3+Light
P9
3
SA-β-gal activity/protein (mg)
P9+Light
P3
2
P3+Light
P5
1
P5+Light
0
HFD
NFD
