# Supplementary material for: Photomodulation alleviates cellular senescence of aging adipose-derived stem cells
Source: Cell Commun Signal. 2023 Jun 19;21:146. doi: 10.1186/s12964-023-01152-x (PMC10278352; doi:10.1186/s12964-023-01152-x)
Supplement: Supplementary file 3 — Additional file 2: Supplemental Table 1. Primers used in qPCR. [file 12964_2023_1152_MOESM2_ESM.docx]

**Supplemental Table 1**

**Primers used in qPCR**

| **Gene** |  | **Forward** | **Reverse** |
| --- | --- | --- | --- |
| 18S |  | CCTGGATACCGCAGCTAGGA | GCGGCGCAATACGAATGCCCC |
| P21 |  | ACTTCCTCTGCCCTGCTGC | GGTCTGCCTCCGTTTTCG |
| P16 |  | ATGGAGTCCGCTGCAGACAG | ATCGGGGTACGACCGAAAG |
| P53 |  | CCCCAGGATGTTGAGGAGTT | TTGAGAAGGGACAAAAGATGACA |
| mTel |  | CGGTTTGTTTGGGTTTGGGTTTGGGTTTGGGTTTGGGTT | GGCTTGCCTTACCCTTACCCTTACCCTTACCCTTACCCT |
| 36B4 |  | ACTGGTCTAGGACCCGAGAAG | TCAATGGTGCCTCTGGAGATT |
